# Annotation and query of tissue microarray data using the NCI Thesaurus

**DOI:** 10.1186/1471-2105-8-296

**Published:** 2007-08-08

**Authors:** Nigam H Shah, Daniel L Rubin, Inigo Espinosa, Kelli Montgomery, Mark A Musen

**Affiliations:** 1 Stanford Medical Informatics, School of Medicine, Stanford University, Stanford, CA 94305, USA; 2 Department of Pathology, School of Medicine, Stanford University, Stanford, CA 94305, USA

## Abstract

**Background:**

The Stanford Tissue Microarray Database (TMAD) is a repository of data serving a consortium of pathologists and biomedical researchers. The tissue samples in TMAD are annotated with multiple free-text fields, specifying the pathological diagnoses for each sample. These text annotations are not structured according to any ontology, making future integration of this resource with other biological and clinical data difficult.

**Results:**

We developed methods to map these annotations to the NCI thesaurus. Using the NCI-T we can effectively represent annotations for about 86% of the samples. We demonstrate how this mapping enables ontology driven integration and querying of tissue microarray data. We have deployed the mapping and ontology driven querying tools at the TMAD site for general use.

**Conclusion:**

We have demonstrated that we can effectively map the diagnosis-related terms describing a sample in TMAD to the NCI-T. The NCI thesaurus terms have a wide coverage and provide terms for about 86% of the samples. In our opinion the NCI thesaurus can facilitate integration of this resource with other biological data.

## Background

Tissue Microarrays allow for the immunohistochemical analysis of large numbers of tissue samples and are used for confirmation of microarray gene-expression results as well as for predictive pathology [1]. A single tissue microarray (TMA) paraffin block can contain as many as 500 different tumors, enabling the screening of thousands of tumor samples for protein expression using a few array sections [2]. Commercial digital-imaging systems can rapidly store thousands of images resulting from such sections. The Stanford Tissue Microarray Database (TMAD) provides a central repository for data from TMA's akin to the Stanford Microarray Database (SMD) for gene expression arrays.

Superficially the datasets generated from TMA and gene expression arrays appear similar in that both are matrix type data and each entry in the matrix provides information about the expression of a biological entity (gene or protein) in a particular sample. However, gene expression arrays query a large number of genes in one sample or patient, whereas Tissue microarrays query a large number of samples/patients for one protein. The key query dimension in TMA data is a tissue sample, rather than a gene. As a result, queries such as 'find all genes that have a function X' get morphed to a query such as 'find all tissue samples that have a particular diagnosis'. Currently, because of the lack of a commonly used ontology to describe the diagnosis or disease state for a given TMA sample in TMAD – analogous to the Gene Ontology for the function of gene products – it is not possible to perform such as query. The lack of an ontology for diagnoses and disease states also limits future integration of this resource with other public genomic scale data, such as gene expression arrays [2,3].

For the purpose of this project, the key challenge was to create consistent ontology/terminology labels for each sample/record in the TMAD that would allow the identification of all samples that are of the same type at a given level of granularity. (e.g., All carcinoma samples versus all Adenocarcinoma in situ of prostate samples, where the former is at a coarser level of granularity). One mechanism of achieving this objective is to map the text-annotations describing the diagnosis of a particular sample to ontology terms that allow us to formulate refined or coarse search criteria [4].

In the current work, we map the text annotations for records in the TMAD to terms from the NCI thesaurus, present the results on the quality of the mapping effort, describe the implementation of the mapping tools on the TMAD website and explain how the mapping enables better querying of the data in TMAD. To the best of our knowledge this is the first work that describes the use of the NCI thesaurus for this purpose.

## Methods

### Overview of data in TMAD

The Tissue Microarray Database (TMAD) contains data from immunohistochemical and in situ hybridization analysis of a large number of tissue samples that were studied with tissue microarrays. The TMAD provides tools for quick upload, storage and retrieval of the TMA images and the analysis of immunohistochemical staining results [5]. Each record, describing the sample donor tissue, in the TMAD contains free-text annotations – entered by the experimenter – for several fields such as the organ system, the source of the sample, the antibody or probe used for staining, and the staining result. Among these fields are up to five diagnosis terms (one principal diagnosis field and four sub diagnosis fields) describing the sample as well as a label for the organ and organ system from which the sample is derived. There are separate tables in the database for keeping track of user logins, experiment details, array constructions etc which are not relevant to the current work.

### The NCI Thesaurus for Annotating TMA Data

Most of the samples in TMAD are cancerous tissue samples and therefore we needed ontologies that provide a broad coverage of the various cancers. The NCI Thesaurus was thus a natural candidate to consider for annotating the samples in TMAD. In previous work, we also included the SNOMED-CT for consideration[6]. However, TMAD would need a license to use the SNOMED-CT in their regular setup as they have non-US based users, therefore, while deploying the final system on TMAD we decided to restrict ourselves to the NCI thesaurus initially.

The NCI Thesaurus is an ontology providing broad coverage of the cancer domain, including cancer-related diseases, findings and abnormalities [7]. In certain areas, such as cancer diseases and combination chemotherapies, the NCI Thesaurus provides the most granular and consistent terminology available. The Thesaurus currently contains over 34,000 concepts, structured into 20 taxonomic trees. It is published under an open content license. The NCI thesaurus can be obtained at . We downloaded version Thesaurus-05.09 g in the tab delimited text format. This text file contains columns for an id, name, parents, synonyms and definition for each NCI thesaurus term. The parents and synonyms columns contain the immediate parents of the term and its synonyms separated by '|' respectively.

However, this format is not optimal for searching parent-child terms rapidly as well as for the purpose of matching against individual synonyms. Therefore we reorganized the thesaurus into three separate tables which we named nci_term, nci_children and nci_synonyms. The nci_term table contains the Thesaurus-05.09 g as is. The nci_children table contains one row for each direct parent-child relationship and the nci_synonyms table contains one row each for each synonym of a term. This reorganization allows us to rapidly search the synonyms of a term as well as identify the parents and child terms of a given term. The tab-delimited files corresponding to these tables as well as the scripts to perform this reorganization are available on request.

### Mapping the TMA annotations to Ontologies

In order to map existing annotations of samples in the TMA database to ontology terms during the test phase [6], we created a database containing the TMAD data, the NCI thesaurus and the SNOMED-CT (derived from UMLS). We used Perl scripts to process the existing descriptions of tissue samples and to generate strings for matching with ontology terms. We identified several heuristics to increase the accuracy of our matches. The Perl scripts used for matching are available from the authors. After several refinements to the matching methods, the final version of the mapping scripts and a copy of the NCI thesaurus are deployed on the TMAD servers so that the text-descriptions of new samples can be processed at regular intervals and the ontology-based annotations can be kept up to date.

### Generation of annotation permutations

Each record in the TMA database has an organ, a diagnosis and four sub diagnosis terms associated with it. For example, a record might contain the entries breast, carcinoma, ductal, <null>, in situ, <null> for the organ (O), diagnosis (d0), sub diagnosis 1 (d1), sub diagnosis 2 (d2), sub diagnosis 3 (d3) and sub diagnosis 4 (d4) fields respectively. We refer to the diagnosis related entries (d0, d1, d2, d3, d4) as a term-set.

We generated all possible permutations of the non-null entries in every term-set. In theory, we would generate over a million permutations for the 10734 records contained in the TMAD at the time of this writing. However, these 10734 records correspond to 1045 unique term-sets, many of which contain fewer than five entries, and in practice we ended up with about twenty thousand permutations. We used the pair of a particular term-set permutation and the associated organ in the search for an ontology term to associate with that TMA record

### Heuristics for increasing match accuracy

When searching the NCI thesaurus, instead of searching for an ontology term for each permutation-organ pair, we first filtered out the non-informative permutations using heuristics that identify such permutations. For example, there are a number of uninformative records where d0 = normal and d1 = 19w which we identify using regular expressions. We also "tweak" a permutation to convert it to the most useful form. For example, if the first word in a permutation is carcinoma and the second word is adeno then flip the order and merge the two words to make the first word to be adenocarcinoma. In another example, if the first word in a permutation is carcinoma and the second word is squamous then flip the order but keep the words separate.

We use such a processed permutation and search for an exact match with a term or a synonym in the NCI thesaurus. If there is no match, then we drop the right most term (similar to right truncation in the UMLSKSS search) and repeat the search. When, during this process, the permutation contains two words or fewer, we add ' of <organ>' to the search string where <organ> is obtained from TMAD.

We also use simple heuristics to weed out bad matches. For example, if a matched term contains uterine but the organ associated with the record is not ovary, uterus, or fallopian tube, then we discard the match. If the matched term contains mouse, then we discard the match.

### Refining heuristics in consultation with pathologists

After initial success reported at AMIA Annual Symposium in 2006, we examined the unmatched records and their corresponding term-sets in collaboration with Inigo Espinosa (pathologist) and Kelly Montgomery (research staff member) from TMAD to refine our heuristics. For example, if the permutation contains 'sertoli leydig' convert it to Sertoli-Leydig. We also added exceptions to our heuristics such as: if the permutation contains two words or fewer and one of the words is 'cholangiocarcinoma' do not add the 'of <organ>' to the search. Such consultations, though time-consuming, turned out to be crucial in achieving high match accuracy.

### Deployment architecture

**Figure 1:**
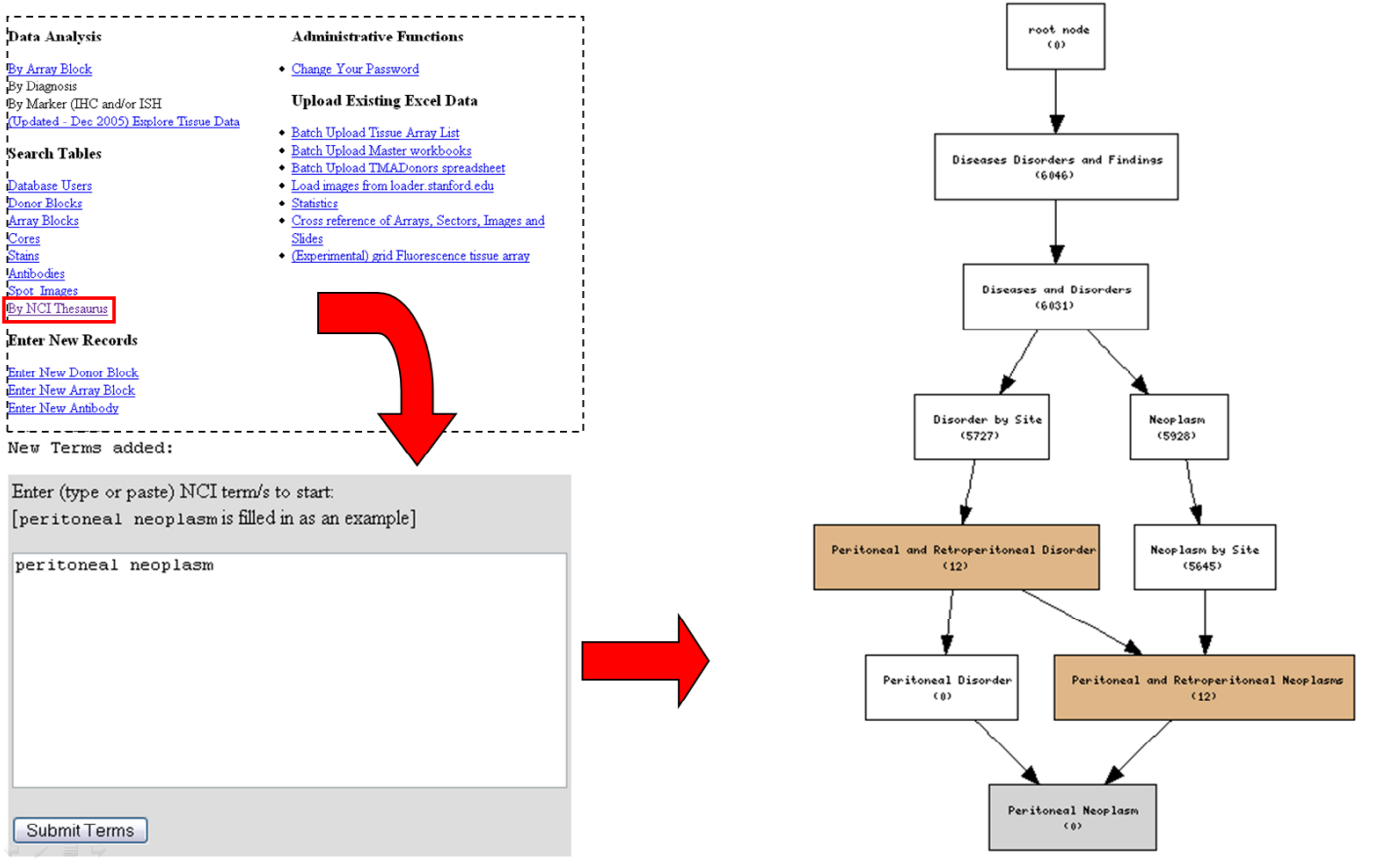
**NCI-thesaurus based browsing interface**. The figure shows our NCI-T based browsing interface. The user begins a query by typing a term in a text box. The same methods that map a sample's description to NCI terms will match the query words to NCI terms. Matched terms and the number of samples corresponding to each term are then presented in a graph view which explained further in the next figure.

**Figure 2:**
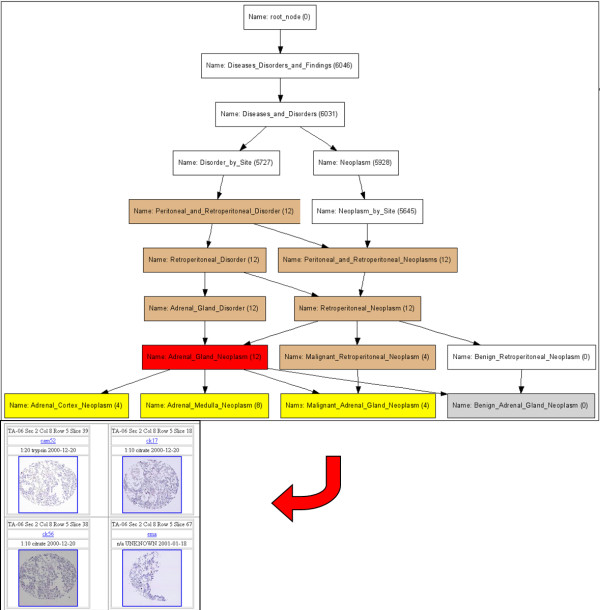
**Details of the NCI-thesaurus based browsing interface**. The figure shows a zoomed in region of the DAG view resulting from clicking on the term Adrenal gland neoplasm as described in the example in the main text. The red node is the term that has been clicked by the user, the yellow nodes are the child terms that have at least one sample in the TMA database assigned to that term, grey nodes are child terms with no corresponding samples in the TMAD and burlywood nodes are parent terms with less than 50 samples. Samples can be retrieved for the selected node.

**Figure 3:**
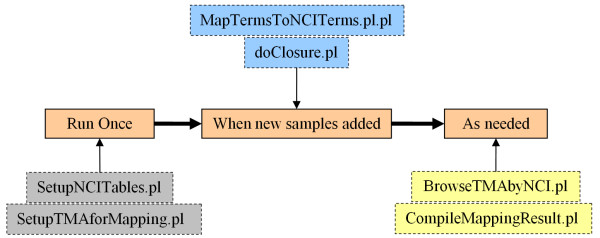
**Overview of the process of annotating TMAD samples with NCI terms**. The figure shows the workflow for setting up TMAD to use NCI thesaurus for annotating tissue microarray samples. The scripts in the grey boxes have to be run once; they create the necessary tables to load the NCI-T in relational form as well as the tables storing the mapping b/w a sample id, its user specified annotation and the matched NCI term. The scripts in the blue boxes are run every time new samples are added to TMAD. The Browsing script (yellow boxes) is run when a user navigates TMAD using the NCI-T terms.

The setup, mapping to NCI terms and browsing are all carried out using modular perl scripts that are used as shown in Figure 3. The scripts are divided into three groups: 1) Setup scripts (SetupNCITables.pl and SetupTMAforMapping.pl) create the necessary tables to store the NCI thesaurus in relational tables as well as create the tables to store the NCI-term annotation of a particular tissue microarray sample. 2) The update scripts (MapTermsToNCITerms.pl, doClosure.pl) are run once after the setup and perform the mapping of user submitted annotation terms to NCI thesaurus terms as well as index the particular tissue sample with the parents of the assigned NCI term. This enables a pheochormocytoma sample to be counted as a retroperitoneal tumor. 3) The browsing script (BrowseTMAbyNCI.pl) drives the interface shown in figures 1 and 2. The directed acyclic graphs are produces using the open source Graphviz library. A utility script, CompileMappingResult.pl, gives summary statistics on the number of mapped and unmapped samples.

## Results

We mapped the term-sets corresponding to the 10734 records from the TMAD, which specified a diagnosis, to ontology terms in the NCI thesaurus. Out of the 1045 distinct term-sets (corresponding to the 10734 samples) we were able to match 902 term-sets to the NCI thesaurus. As mentioned before, in our initial work we had also mapped the term-sets to SNOMED-CT terms. TMAD would need a license to use the SNOMED-CT in their regular setup because they have non-US based users; therefore, while deploying the final system on TMAD we restrict ourselves to the NCI thesaurus only. In total we were able to map 9283 (86%) of records in TMAD to one or more terms from NCI-T.

### Example Matches to the NCI Thesaurus

The NCI thesaurus has a wide coverage in providing matching terms for associating with diagnosis term-sets from the TMAD. Term-sets for a total of 9283 records could be mapped to one or more terms from the NCI thesaurus. The type (and the granularity level) of the ontology terms that matched a given term-set varied over a wide range. For example, there are records where the term-set contained just four characters, such as MMMT (of ovary) and matched a very specific ontology term such as 'Malignant_Mixed_Mesodermal_Mullerian_Tumor'. At the same time, there are records where the term-set was highly descriptive such as carcinoma adeno intraductal (of prostate) which matched an ontology term such as 'Prostate_Ductal_Adenocarcinoma' and records where the term-set was highly descriptive such as carcinoma transitional cell in situ (of bladder) which matched two ontology terms – 'Stage_0_Transitional_Cell_Carcinoma' and 'Bladder_Carcinoma' because no single term existed that would capture all the information.

### Evaluation of Precision and Recall

In our work, we mapped 9283 records from TMAD to one or more ontology terms; it is extremely time consuming for a domain expert to evaluate every matched term manually. Therefore, we devised a sampling strategy where from the records that had a match, we randomly selected 50 rows comprising a distinct term-set, the associated organ, and the matched ontology term to determine the percentage of matches that were appropriate (true positive) or inappropriate (false positive) on manual inspection. We do not consider high-level matches such as *sarcoma clear cell (of soft tissue) *– which matches 'Sarcoma of the soft tissue and bone' – and *mucinous neoplasm papillary intraductal (of pancreas) *– which matches 'Mucinous Neoplasm' – to be appropriate hits in this evaluation. We repeated this procedure 3 times. The results of this exercise are presented in Table 1. 14% of the reported hits were deemed to be false positives, giving a precision of 86%.

**Table 1:** Precision calculation for three samples from the matched records

	True Positive	False Positive
Set-1	44	6
Set-2	42	8
Set-3	43	7

Total	129	21

Average (%)	43.0 (86%)	7.0 (14%)

For 1451 rows, corresponding to 143 term-sets (13.3%), we did not have a match with any NCI term. Potentially all of these are false negatives putting a lower bound on the recall to be 86.7%. We adopted a similar sampling strategy to select 3 sets of 50 records where no match was found. A domain expert then examined the NCI thesaurus attempting to find a match for those records. The results are presented in Table 2.

**Table 2:** False negatives for three samples from the unmatched records

	True negative	False negative
Set-1	36	14
Set-2	31	19
Set-3	31	19

Total	98	52

Average (%)	32.6 (66%)	17.4 (34%)

We were manually able to find a match for 34% of those records, meaning that for these records, a true match did exist in the NCI thesaurus that our methods were unable to find. Examples of such record is *carcinoma conventional granular pauci cellular area (of kidney) *where a match exists in the NCI thesaurus as *conventional renal cell carcinoma or clear cell renal cell carcinoma *This fraction of the unmatched records are false negatives, giving us an estimated false negative rate of about 4.52 % and an estimated recall of 95%.

For the remaining records, no good match exists and they are true negatives. Among these true negatives, many term-sets did not describe any cancer and consisted of words such as *proximal area history of lymphoma (of Colon)*, *mesial schelorsis epilepsy (of brain) *and *no cancer in prostate*. It is not surprising that these term-sets did not match anything in the NCI thesaurus because these are not cancer related terms. In some cases the term-sets were contradictory such as the diagnosis being *leiomyosarcoma *but the organ specified as *skeletal muscle*, instead of *smooth muscle*. There are several records that have words like *10w *as the diagnosis, these are skipped during matching and considered as true negatives.

We note that Metamap is considered the gold standard in mapping medical text to UMLS concepts [8-10]. However, Butte et al have previously applied Metamap (GenoText) to determine the phenotypic and experimental context from text annotations of GEO experiments [11]. They report that text-parsing using Metamap is still an inefficient method to extract value from text annotations of genomic datasets [12]. In subsequent work they have explored much simpler methods such as ours and report that simple heuristics outperform Metamap indicating that the complexity of Metamap may not be essential for processing text annotations of genomic datasets[10].

### Mapping to NCI enables better querying/analysis of TMA data

Once the mapping is accomplished, the simple assignment of the ontology terms to tissue samples is still not immediately useful to the end-user unless these ontology terms are used to drive specialized query-interfaces. Plain keyword-based querying of ontology terms is not very useful. Therefore, we are developing a querying and browsing interface (shown in Figure 1) where the user starts with a term, visualizes the 'neighboring' ontology terms of that term in a DAG view and browses up, and down the ontology-term hierarchy to identify a term that is at the right level of granularity. This term then can be used to pull out all the TMAD records that are associated with that term or its child-terms. This interface is similar in spirit to Amigo, which is used for browsing the gene ontology (and GO annotations) at the gene ontology consortium's website [13].

Using this interface, a user can query a term such as "peritoneal neoplasm" to find that there are no samples corresponding to that particular term, but at the same time, the user can see that there are 12 samples corresponding to the parent term (peritoneal or retroperitoneal neoplasm), and can click on the retroperitoneal neoplasm term to find that these are adrenal gland neoplasms and that four of them are from the medulla, and that eight are from the cortex of which four are malignant (Figure 2).

The user can then choose to retrieve all 12 samples corresponding to retroperitoneal neoplasm or retrieve the samples corresponding to specific terms such as adrenal cortex neoplasm. Having annotations based on NCI-T also enables formulation of queries (by browsing) to pose requests such as: 1) Show all retroperitoneal tumors 2) How many skin neoplasms does TMAD have? 3) What subtypes of malignant abdominal neoplasms does TMAD have? These queries could not be posed with the standard keyword based search interface.

## Discussion

Currently there is a lot of effort directed towards classification of tumors using gene expression data[14]. There are significant efforts directed towards creating centralized repositories of protein expression patterns observed on tissue microarrays as well as the underlying images such as the Stanford tissue microarray database and Tissue array database at MD Anderson cancer.

DNA microarrays and tissue microarrays query two orthogonal dimensions of the data. The former query the expression of many genes for one patient in one experiment whereas the latter query many patients for changes in expression of one protein in a single experiment. The results from such studies have to be integrated with each other and with traditional histopathological diagnosis methods in order to form testable hypotheses about the underlying molecular mechanisms which determine prognosis of a particular cancer. However, little attention is being paid to the problem of developing mechanisms to *integrate *the results from these two complementary data types. Recent reviews have suggested that it is essential to address this issue and synchronize the analysis, interpretation and data standards for these data [15]

**Figure 4:**
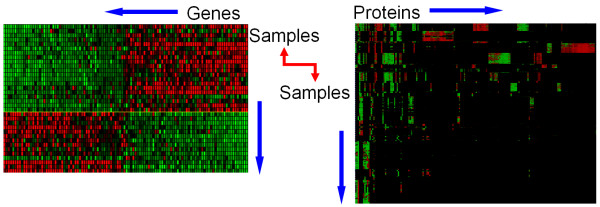
**Schematic showing the need to integrate annotations of tissue samples**. The figure shows a schematic of the need for a specific mechanism to identify relevant *samples *in tissue and gene expression databases to perform integrative analyses. The correspondence denoted by the red arrow is hard to establish with free text sample descriptions.

We believe such integration can be achieved via the use of explicit ontologies to link specific histological features, both morphological anomalies as well as IHC results, to gene expression changes in specific biological processes and pathways. The mouse pathology ontology is a significant first step in this direction and can potentially allow systematic integration of expression data with histopathology slides[16]. However, no such equivalent exists for describing human histopathology and IHC studies as well as for combining their results with analyses of gene expression studies. The current work – where we have automatically mapped approximately 86% of diagnoses-related annotations for the samples in the Stanford TMAD to terms in ontologies – is a step in that direction. Though detailed morphological annotations might not be currently feasible, simple annotations using terms from the NCI thesaurus provide a specific mechanism to identify relevant *samples *in tissue and gene expression databases to perform integrative analyses (Figure 4).

It is possible to perform a similar procedure for microarray data sets corresponding to disease samples in the Stanford Microarray Database (SMD) and/or the Gene Expression Omnibus (GEO). If the microarray samples are similarly annotated with the diseases and/or tissue they are derived from, that will enable integrated analysis of protein and mRNA expression datasets. In follow up efforts to the current work, we have applied our methods to text annotations of GEO and are able to identify disease related experiments with high precision and recall as well as identify candidate datasets for integrated analysis of protein and mRNA expression [17]. We note that though the coverage of NCI-T was adequate for the current work, it might not be so for other situations. Therefore we have made our methods available as a PERL module that can be used to annotate samples using terms from any ontology that is in the UMLS.

This also presents an exciting research opportunity for specific cancer types, such as lung cancer, where integration of these data is needed for better tumor profiling [18]. Such integration, can drive further research to develop formal guidelines for classifying and profiling tumors as well as making predictive pathology recommendations for customized chemotherapeutic intervention based on constraints defined on gene expression and histopathological phenotypes [1,3,19].

## Conclusion

We have demonstrated that we can effectively map the diagnosis-related terms describing a sample in TMAD to the NCI-T. The NCI thesaurus terms have a wide coverage and provide terms for about 86% of the samples. We deployed a set of mapping scripts at TMAD that will update the ontology annotations as new records are entered, ensuring that the structured annotations using NCIT terms are always up to date. We have developed and deployed a graphical interface to browse the sample collection in TMAD using the NCI thesaurus terms and their hierarchical organization. We described how such a mapping allows a rich querying facility and offers the ability to identify "similar" or "related" tissue microarray samples, even though they may be described by different terms. For example, the four neoplasms of the adrenal medulla and eight neoplasms of the adrenal cortex (four of which are malignant) are all related to each other by the fact that they are all retroperitoneal neoplasms. Finally, we note that such ontology based annotation can enable better data integration across diverse repositories similar to how GO has enabled such integration across model organism databases.

## Availability and Requirements

Source code for both, the mapping scripts and the browsing interface is available as additional files [see Additional files 1 and 2]. The main functionality of the mapping scripts – of generating permutations of text-annotations and mapping them to UMLS terms – is also made available packaged as a PERL module (called UMLSQuery) from [20].

**Operating system(s): **Windows XP, Linux;

**Programming language: **PERL;

**Other requirements: **PERL 5.6 or higher, Graphviz 1.2 or higher;

**License: **GNU GPL;

**Any restrictions for non-academics: **none.

## Authors' contributions

NS conceived of the project, developed the mapping method and wrote the code. DL, IE, KM participated in the evaluation of the method. IE, KM helped refine the match heuristics. All authors have read and approve of the manuscript.

## Supplementary Material

Additional file 1 Mapping Code. This zipped file contains the Perl scripts to perform the mapping of the TMA annotations as described in the methods section. Click here for file

Additional file 2 Browsing Code. This zipped file contains the Perl scripts to create the browsing interface shown in Figure 1. Click here for file
